# Salience Network and Depressive Severities in Parkinson’s Disease with Mild Cognitive Impairment: A Structural Covariance Network Analysis

**DOI:** 10.3389/fnagi.2017.00417

**Published:** 2018-01-10

**Authors:** Ya-Ting Chang, Cheng-Hsien Lu, Ming-Kung Wu, Shih-Wei Hsu, Chi-Wei Huang, Wen-Neng Chang, Chia-Yi Lien, Jun-Jun Lee, Chiung-Chih Chang

**Affiliations:** ^1^Department of Neurology, Kaohsiung Chang Gung Memorial Hospital, Chang Gung University College of Medicine, Kaohsiung, Taiwan; ^2^Department of Psychiatry, Kaohsiung Chang Gung Memorial Hospital, Chang Gung University College of Medicine, Kaohsiung, Taiwan; ^3^Department of Radiology, Kaohsiung Chang Gung Memorial Hospital, Chang Gung University College of Medicine, Kaohsiung, Taiwan

**Keywords:** brain imaging, cognition, depression, mood disorders, neuroimaging

## Abstract

**Purpose:** In Parkinson’s disease with mild cognitive impairment (PD-MCI), we investigated the clinical significance of salience network (SN) in depression and cognitive performance.

**Methods:** Seventy seven PD-MCI patients that fulfilled multi-domain and non-amnestic subtype were included. Gray matter structural covariance networks were constructed by 3D T1-magnetic resonance imaging and seed based analysis. The patients were divided into two groups by psychiatric interviews and screening of Geriatric Depression Scale (GDS): PD-MCI with depression (PD-MCI-D) or without depression (PD-MCI-ND). The seed or peak cluster volume, or the significant differences in the regression slopes in each seed-peak cluster correlation, were used to evaluate the significance with the neurobehavioral scores.

**Results:** This study is the first to demonstrate that the PD-MCI-ND group presented a larger number of voxels of structural covariance in SN than the PD-MCI-D group. The right fronto-insular seed volumes and the peak cluster of left lingual gyrus showed significant inverse correlation with the Geriatric Depression Scale (GDS; *r* = -0.231, *P* = 0.046).

**Conclusions:** This study is the first to validate the clinical significance of the SN in PD-MCI-D. The right insular seed value and the SN correlated with the severity of depression in PD-MCI.

## Introduction

Although Parkinson’s disease with mild cognitive impairment (PD-MCI) is conceptualized as a transitional state between normal cognition and dementia, it is a major predictor for the conversion of dementia (Domellof et al., 2015). The quality of life in patients with PD-MCI is limited (Reginold et al., 2013), and the presence of depression is one of the most common non-motor symptoms (Burn, 2002; Aarsland et al., 2012). PD-MCI with depression (PD-MCI-D) has also been identified as a risk factor for dementia (Modrego and Ferrandez, 2004; Janvin et al., 2006), and the use of cholinesterase inhibitors has been reported to delay the cognitive progression (Lu et al., 2009). For PD-MCI, the natural history of PD-MCI-D does not parallel that of motor symptoms, suggesting the distinctive pathophysiological mechanism (Brown and Jahanshahi, 1995). In this context, it is of clinical relevance to explore the neural basis of PD-MCI-D.

In Parkinson’s disease, the salience network (SN) has been implicated in emotional processing and depression (Remy et al., 2005). Several limbic structures were reported in SN that included the insula and anterior cingulate cortex (ACC) (Seeley et al., 2007; Bressler and Menon, 2010). The reduced Blood oxygenation level dependent (BOLD) response and reduced gray matter (GM) volume within the SN in Parkinson’s disease have been reported to mediate the emotional valence (Cardoso et al., 2009; Kostic et al., 2010). In PD-MCI, the GM atrophy of SN (Beyer et al., 2007; Song et al., 2011) showed mixed evidence for predicting the conversion to dementia (Aybek et al., 2009; Jokinen et al., 2009; Martin et al., 2009). Despite the solid evidence of SN in PD with depression (Beyer et al., 2007; Kostic et al., 2010; Song et al., 2011), whether the changes in the SN also mediate the depression state in PD-MCI is not fully investigated.

The current neuronal scaffold of neurodegenerative diseases emphasizes the pathological protein accumulation within the large-scale networks that are anatomically distinctive (Seeley et al., 2009). The application of structural covariance networks (SCNs) has been supported by recent research in that highly related regions may show covariance in morphometric characteristics. SCN patterns have been shown to be associated with structural or functional connectivity while the structural covariance strength often reflects how close two interconnected hubs interact (Alexander-Bloch et al., 2013). The SCN of SN included the insular seed and peak clusters in the prefrontal cortex, ACC, left angular gyrus, and medial and lateral temporal cortex (Clos et al., 2014). The covariance strength between seed and peak clusters has been found to associated with social cognition, reward, explicit memory, and negative emotion (Zaki et al., 2007; Jabbi and Keysers, 2008; Clos et al., 2014). The SCN might serve as a potential model for understanding whether the SN was involved in depressive state or severity in PD-MCI.

To date, there is no literature for depressive network analysis in PD-MCI. This study explored whether the SN may signify PD-MCI-D. Meanwhile, we explored whether the seed or peak cluster volume or the seed-peak covariance strength may determine the cognitive performance or depressive scores in patients with PD-MCI.

## Materials and Methods

### Subjects

The study patients were treated at the Department of Neurology, Kaohsiung Chang Gung Memorial Hospital. This study was approved by the Chang Gung Memorial Hospital Ethics Committee. A total of 89 subjects with PD-MCI (48 male and 41 female subjects) were included after the consensus of a panel composed of neurologists, neuropsychologists, neuroradiologists, psychiatrists, and experts in nuclear medicine (Huang et al., 2015). PD-MCI is diagnosed according to Movement Disorder Society Task Force criteria (Litvan et al., 2012) and the result of formal neuropsychological testing (Chang et al., 2008, 2009) that the cognitive impairment is not sufficient to interfere with functional independence (Litvan et al., 2012). As there were clinical heterogeneities among patients with PD-MCI, only those with multiple domains non-amnestic presentations were included (*n* = 77). The diagnosis of depression was carried out by means of a half-hour structured interview by a psychiatrist according to the Diagnostic and Statistical Manual of Mental Disorders, Fourth Edition (DSM-IV) criteria (Zimmerman et al., 2011). A total of 35 patients were in the PD-MCI-D group, and 42 were in the PD-MCI without depression (PD-MCI-ND) group. All participants were matched for age and years of education and patients were matched for disease severity as measured by the Unified Parkinson Disease Rating Scale III (UPDRS-III) and Hoehn and Yahr scale. A voluntary comparison normal control (NC) group, who had no underlying neurological or psychiatric disorders, was recruited from outpatient neurological and geriatrics clinics.

### Clinical and Neurobehavioral Assessments

We used a comprehensive battery of tests to assess the cognitive ability of all participants. The Mini-Mental State Examination (MMSE) assesses the general intellectual function. The episodic memory was assessed by the Chinese version verbal learning test (CVVLT) using a 9-word list with fixed order over 4 learning trials (Chang et al., 2016). The scores after a 30-s (CVVLT-30 s) and 10-min delay (CVVLT-10 m) were recorded. The semantic verbal fluency tests included the free generations of four categories (animal, fruit, town, and transportation), each for 1 min. The Visual Object and Space Perception Battery (VOSP) and the copy of modified Rey–Osterrieth complex figure and pentagons were used to assess the visual-spatial abilities (Rapport et al., 1998). The subjects’ frontal lobe function was assessed using digit-forward, digit-backward, Stroop interference (Amieva et al., 2004), and modified Trails B tests (Reitan, 1955).

All the patients with PD-MCI completed the Chinese version of the 15-item Geriatric Depression Scales (GDS-15) (Spreen and Strauss, 1998). For illiterate subjects, the scale was read by the interviewers without any comment, and the subjects were asked to choose one of the answers. All patients with PD-MCI-D, previously confirmed by the psychiatrist, showed a higher GDS screening score (**Table 1**) and all were higher than the cutoff value (Marc et al., 2008).

**Table 1:** Demographic data of patients with PD-MCI and normal controls.

	Normal controls	PD-MCI	PD-MCI-ND	PD-MCI-D
Male/Female	14/13	41/36	20/22	21/14
Age (year-old)	63.4 ± 8.1	64.4 ± 10.8	65.8 ± 9.8	62.7 ± 11.8
Education (year)	9.6 ± 2.7	8.9 ± 4.1	8.6 ± 4.0	9.3 ± 4.3
Hoehn and Yahr scale	–		1.8 ± 0.9	1.6 ± 0.7
UPDRS	–		30.8 ± 20.0	27.1 ± 14.7
MMSE	27.9 ± 2.5	25.9 ± 3.8a	25.9 ± 3.7a	25.8 ± 3.9a
Memory test				
CVVLT-30 s	7.3 ± 1.5	6.52 ± 1.8	6.6 ± 1.7	6.5 ± 2.1
CVVLT-10 min	6.7 ± 1.8	6.1 ± 2.0	6.3 ± 2.0	5.9 ± 1.9
Visuospatial function				
Modified R-O copy	16.5 ± 2.2	16.0 ± 2.5	15.8 ± 3.0	16.3 ± 1.7
VOSP	7.9 ± 2.7	7.4 ± 2.8	7.3 ± 1.1	7.1 ± 1.6
Frontal lobe function				
Digital forward	7.9 ± 1.1	7.3 ± 1.4a	7.4 ± 1.1	7.1 ± 1.6a
Digital backward	4.7 ± 1.3	3.9 ± 1.5a	3.8 ± 1.5a	4.0 ± 1.4
Stroop test	36.3 ± 13.7	30.2 ± 13.8	31.1 ± 15.0	29.1 ± 12.5a
Trail making test	12.3 ± 3.4	10.6 ± 4.4	11.4 ± 4.1	9.7 ± 4.6a
Semantic verbal fluency test				
Animal	15.7 ± 4.9	13.5 ± 4.6a	14.2 ± 4.8	12.7 ± 4.3a
Fruit	12.7 ± 3.4	11.3 ± 4.6	11.2 ± 2.6a	11.4 ± 6.3
Town	16.0 ± 7.2	11.7 ± 6.3a	11.4 ± 6.1a	12.1 ± 6.7a
Transportation	9.5 ± 3.2	7.6 ± 2.7a	7.8 ± 2.3a	7.3 ± 3.2a
GDS	2.9 ± 3.2	5.1 ± 3.9a	2.1 ± 1.3	8.6 ± 3.0ab

*^a^*P* < 0.05, compared to normal controls. ^b^*P* < 0.05, compared to PD-MCI-ND. CVVLT, Chinese version of the Verbal Learning Test; GDS, Geriatric Depression Scale; MMSE, Mini-Mental State Examination; PD-MCI, Parkinson’s Disease with mild cognitive impairment; PD-MCI-ND, PD-MCI without depression; PD-MCI-D, PD-MCI with depression; R-O, Rey–Osterrieth; UPDRS, Unified Parkinson’s Disease Rating Scale; VOSP, Visual Object and Space Perception Battery.*

### Magnetic Resonance Imaging (MRI) Acquisition

The T1-weighted sequence, inversion-recovery-prepared, three-dimensional, spoiled, gradient-recalled acquisition in steady-state sequences were acquired using the GE 3T Signa Excite scanner (GE Medical System, Milwaukee, WI, United States) with the following parameters: repetition time/inversion time of 8,600 ms/450 ms, 240 mm × 240 mm field of view, and 1-mm slice thickness.

### MRI Preprocessing

Structural images were preprocessed using voxel-based morphometry (VBM) implemented with Statistical Parametric Mapping 8 software^1^ running under MATLAB 7.9 (MathWorks, Natick, MA, United States). VBM is a whole-brain, unbiased, and semi-automated technique. First, structural images were normalized to the Montreal Neurological Institute stereotactic space and then segmented to extract the GM. Using diffeomorphic anatomical registration via the exponentiated lie algebra approach, related tissue segments were used to create a custom template. The resulting GM images were finally smoothed with an 8-mm isotropic Gaussian kernel (Mechelli et al., 2004).

### Statistical Analysis

Voxel-based morphometry was used to investigate the atrophic patterns among the three clinical groups with specific T contrasts as follows: NC > PD-MCI-ND, NC > PD-MCI-D, PD-MCI-D > PD-MCI-ND or PD-MCI-D < PD-MCI-ND. The threshold for the *t*-test parametric maps was corrected for multiple comparisons using the family-wise error (FWE) correction and with the significance threshold at *P* < 0.001 and cluster size > 100 voxels.

We used seed-based analysis (Lin et al., 2016) to construct the SCN. Regional densities were extracted from the 4-mm radius sphere of the right fronto-insula seed (*x* = 36, *y* = 18, *z* = 4) (Seeley et al., 2007; Menon, 2015) and used to model the regional densities in all voxels of the preprocessed GM segments. The PD-MCI-D and PD-MCI-ND groups were separately modeled. For each group, the specific contrast was set to identify voxels (peak clusters) that showed significant positive correlations between the right insular seed, represented physiologically as “structural associations” (Montembeault et al., 2016). Significant clusters were selected with Family-Wise Error rate (FWE) correction in Bonferroni method and a *P*-value less than 0.001. Only clusters with more than 100 voxels were chosen.

For the group interaction test between the PD-MCI-D and PD-MCI-ND groups in the SN structural associations, statistical contrasts were set to identify seed-peak cluster voxels that expressed differences in the regression slopes. For the difference in the structural association, T contrasts were established to map the voxels that expressed stronger structural associations in the PD-MCI-ND group. The threshold for the resulting statistical parametric maps was established as FWE-corrected for multiple comparisons at *P* < 0.05. Furthermore, voxels showing significant differences in the regression slopes in each seed-peak cluster correlation were compared. Then, a 4-mm radius sphere was placed on these peak voxels, and the GM volume was used to evaluate the clinical significance of these regions. Neurocognitive test scores were the dependent variable in the linear regression model with the seed or peak voxel volume as the predictor, and possible covariates such as GDS scores, age, and education were adjusted. GDS scores were also a dependent variable in the linear regression model with the seed or peak voxel volume as the predictor, and covariates of age and education were adjusted.

Clinical and laboratory data were expressed as mean ± standard deviation. Analysis of variance with Bonferroni correction for multiple comparisons was used to compare continuous variables among the NC, PD-MCI-D and PD-MCI-ND. All statistical analyses were conducted using SPSS software (SPSS version 22 for Windows^®^, SPSS Inc., Chicago, IL, United States). Statistical significance was set at *p* < 0.05.

## Results

### Demographic and Clinical Characteristics

Seventy seven patients with PD-MCI (35 with PD-MCI-D and 42 with PD-MCI-ND) and 27 NC matched for age, gender, and educational levels completed the study (**Table 1**). The PD-MCI was lower than the NC in MMSE scores (*p* = 0.003), scores in digital forward and backward, lower semantic verbal fluency scores and higher GDS than NC (*P* < 0.05). Meanwhile, the PD-MCI-D subgroup showed significantly lower scores in the frontal lobe function and semantic verbal fluency tests than NC group (*P* < 0.05). Except for the GDS scores, the comparisons of PD-MCI-D and PD-MCI-ND groups, however, were not significant (*P* > 0.05).

### Brain Volume Atrophic Patterns

**FIGURE 1:**
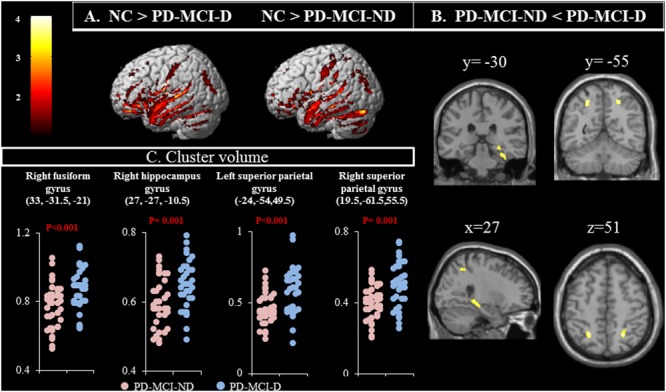
**(A)** Contrast maps of volume changes depicting the brain areas with a significant difference in the gray matter volume in normal controls (NC) vs. patients with Parkinson’s disease-mild cognitive impairment without depression (PD-MCI-ND) and vs. PD-MCI with depression (PD-MCI-D), using voxel-based morphometric methods. **(B)** Contrast maps between PD-MCI-ND and PD-MCI-D. *Z*-statistic maps [*P* < 0.001, corrected for a family-wise error (FWE) and extended cluster voxels > 100]. **(C)** Plot of significant difference between PD-MCI-D and PD-MCI-ND in cluster volume.

Compared with controls, both the PD-MCI-ND and PD-MCI-ND (**Figure 1A**) groups showed atrophy in the bilateral temporal, frontal, and occipital lobes, and the right cerebellum. The right superior occipital and frontal gyri and the left supramarginal gyrus were highlighted for the difference in regional brain volume observed in the PD-MCI-D and PD-MCI-ND groups when compared with the NC group (*p* < 0.001). The differences in the brain volumes between the PD-MCI-ND and PD-MCI-D groups were observed in the right fusiform gyrus, right hippocampus, and bilateral superior parietal gyri (*p* < 0.001; **Figures 1B,C**). The details of the brain areas with significant volumetric differences are listed in **Table 2**.

**Table 2:** Voxel-based morphometry (VBM) difference among normal controls and patients with PD-MCI-ND and PD-MCI-D.

	*x*	*y*	*z*	*T*-score	Voxels
**NC > PD-MCI-ND**					
Right temporal lobe	37.5	–13.5	–12	29.1729	25398
Right cerebellum	30	–27	–31.5	15.0927	7230
Left frontal lobe	–21	48	–21	9.7238	120
Right middle temporal gyrus	58.5	–6	–15	7.4086	146
Left middle occipital gyrus	–34.5	–73.5	25.5	12.7509	268
Right calcarine gyrus	16.5	–70.5	15	11.2434	166
Right middle occipital gyrus	37.5	–67.5	31.5	12.0893	253
Right superior occipital gyrus	22.5	–64.5	31.5	11.8185	169
Left supramarginal gyrus	–49.5	–34.5	33	13.149	100
Right supramarginal gyrus	51	–30	36	13.0843	135
Left middle frontal gyrus	–24	31.5	42	9.1489	143
Right middle frontal gyrus	25.5	19.5	52.5	7.6016	142
**NC > PD-MCI-D**					
Right temporal lobe	37.5	–13.5	–12	28.6554	25033
Right cerebellum	30	–27	–31.5	14.794	6511
Left frontal lobe	–21	48	–21	9.4818	121
Right middle temporal gyrus	58.5	–6	–15	7.2187	127
Right middle frontal gyrus	34.5	40.5	15	7.4607	100
Left middle occipital gyrus	–34.5	–73.5	25.5	12.4705	247
Right calcarine gyrus	16.5	–70.5	15	11.0682	163
Right middle occipital gyrus	37.5	–67.5	31.5	11.8221	217
Right superior occipital gyrus	22.5	–64.5	31.5	11.5668	163
Right supramarginal gyrus	51	–30	36	12.7912	131
Left middle frontal gyrus	–24	31.5	42	8.9764	141
Right superior frontal gyrus	25.5	21	51	7.502	142
**PD-MCI-D > PD-MCI-ND**					
Right fusiform gyrus	33	–31.5	–21	3.9212	103
Right hippocampus gyrus	27	–27	–10.5	3.7796	108
Left superior parietal gyrus	–24	–54	49.5	4.3339	122
Right superior parietal gyrus	19.5	–61.5	55.5	4.6819	138

*All *Z*-scores are significant difference at *P* < 0.001 and cluster > 100 voxels. NC, normal controls; PD-MCI-ND, Parkinson’s Disease with mild cognitive impairment with depression; PD-MCI-ND, Parkinson’s Disease with mild cognitive impairment without depression; VBM, voxel-based morphometry.*

The relationship between more atrophic clusters in the PD-MCI-ND group with the selected cognitive test was further explored. After controlling for age and education, the GDS was positively correlated with volume in the right fusiform gyrus (*r* = 0.351, *P* = 0.002) and the left superior parietal lobe (*r* = 0.368, *P* = 0.001). The VOSP was positively correlated with volume in right hippocampus (*r* = 0.305, *P* = 0.008) and the right superior parietal lobe (*r* = 0.332, *P* = 0.004).

### Patterns of Structural Associations in the PD-MCI-ND and PD-MCI-D Groups

**FIGURE 2:**
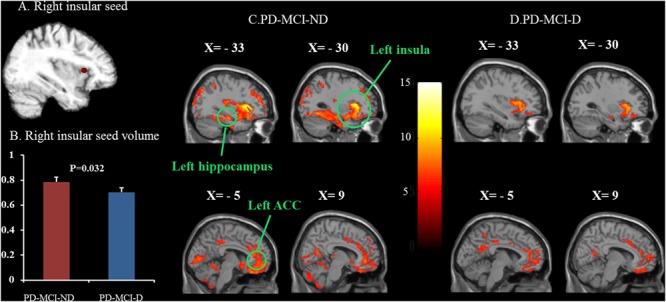
Statistical maps depicting brain areas in which the gray matter intensity covaried with the frontoinsular seed **(A)**, seed volume **(B)**, and structural covariance networks in the PD-MCI-ND and PD-MCI-D groups **(C,D)**. There was significant decrease in the seed volume in the PD-MCI-D group compared with the PD-MCI-ND group (*P* = 0.032). *Z*-statistic maps (*P* < 0.001, corrected with an FWE and extended cluster voxels > 100). The images are displayed on a standard brain render.

The representative right insular seed is shown in **Figure 2A**, and the seed volume was significantly higher in the PD-MCI-ND group (**Figure 2B**). Using the seed and correlation analysis, the spatial patterns of SN in the PD-MCI-ND (**Figure 2C**) and PD-MCI-D (**Figure 2D**) groups share similar patterns (**Table 3**). However, the PD-MCI-ND group presented a larger number of voxels (the insula, hippocampus, and ACC: 44934 voxels) than PD-MCI-D (the insula, hippocampus, and ACC: 6421 voxels).

**Table 3:** Seed-based structural covariance network.

	*x*	*y*	*z*	*Z*-score	Voxels
**PD-MCI-ND**					
Right Insula	36	18	4.5	39.1912	44934
Left inferior parietal	–31.5	–73.5	40.5	7.0032	2060
Left middle cingulate cortex	–1.5	–34.5	39	5.5926	369
Right middle cingulate cortex	10.5	3	43.5	4.6547	127
Right inferior temporal	48	3	–36	4.6695	147
Right middle temporal	52.5	–64.5	3	7.0035	2524
Left superior temporal	–52.5	–9	1.5	4.3635	179
Left inferior frontal	–42	6	25.5	4.7499	204
**PD-MCI-D**					
Left medial orbitofrontal	–7.5	52.5	–3	6.2846	2901
Right middle temporal	57	4.5	–22.5	6.468	126
Left inferior temporal	–60	–25.5	–19.5	6.1856	266
Left hippocampus	–15	–9	–15	5.37	152
Right insula	36	18	6	23.5801	3886
Right hippocampus	37.5	–21	–16.5	4.232	105
Left insula	–37.5	9	3	9.1382	2164
Right anterior cingulate cortex	9	39	18	4.8467	114
Left precuneus	–10.5	–54	34.5	6.1958	1100
Right middle cingulate cortex	6	22.5	31.5	4.8313	496
Left middle cingulate cortex	–6	–22.5	37.5	5.2058	136

*All *Z*-scores are significantly correlated with seed volume at *P* < 0.001 and cluster > 100 voxels. PD-MCI-D, Parkinson’s Disease with mild cognitive impairment with depression; PD-MCI-ND, PD-MCI without depression.*

### Covariance Strength Interactions between the Two PD-MCI Groups

**FIGURE 3:**
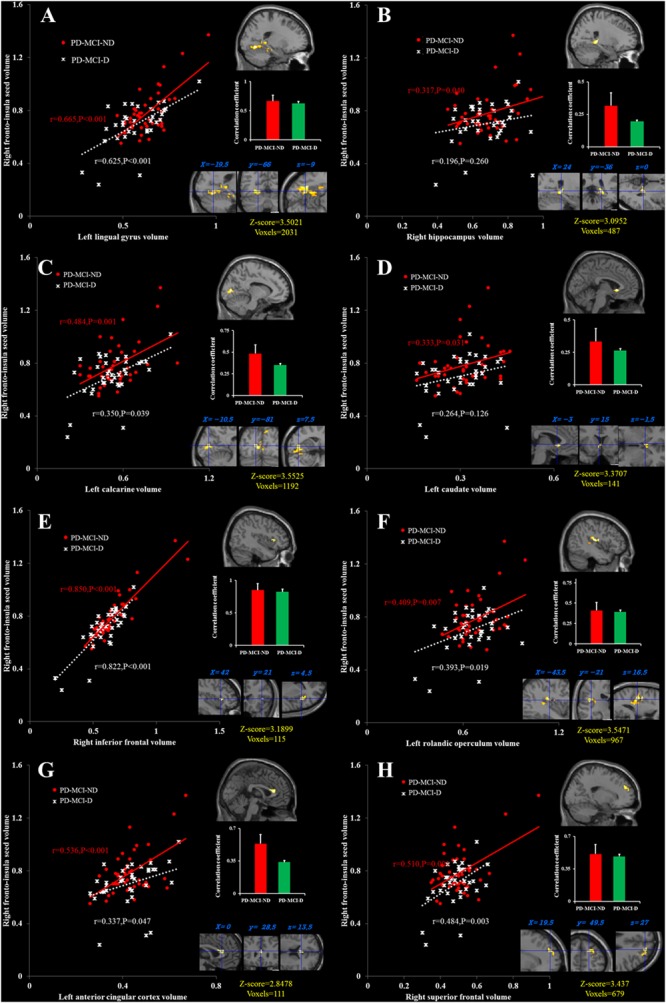
Peak voxels that expressed decreased structural association in the PD-MCI-D group compared with the PD-MCI-ND group, and correlations between the gray matter volumes extracted from a 4-mm radius sphere centered on the frontoinsular seed and a 4-mm radius sphere centered on these peak voxels, including the left lingual gyrus **(A)**, the right hippocampus **(B)**, the left calcarine **(C)**, the left caudate **(D)**, the right inferior frontal cortex **(E)**, the left rolandic operculum **(F)**, the left anterior cingulate cortex, **(G)** and the right superior frontal cortex **(H)**. Red dots represent PD-MCI-ND, and white crosses represent PD-MCI-D.

There were eight clusters that showed covariance strength interactions between the two PD-MCI groups (**Figures 3A–H**), all showing stronger covariance coefficients in the PD-MCI-ND group. These included the left ACC, left calcarine, left caudate, right fronto-insula cortex, right hippocampus, right inferior frontal gyrus, left lingual gyrus volume, left rolandic operculum, and right superior frontal lobe (**Figure 3**).

### Seed or Peak Cluster Volumes and Relationships with Neurobehavioral Scores

**FIGURE 4:**
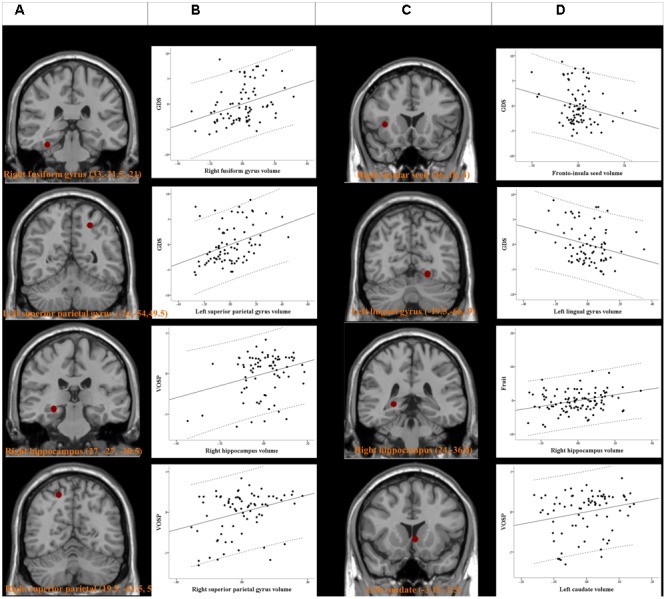
Peak voxels of significant difference in the gray matter volume between PD-MCI-ND and PD-MCI with depression (PD-MCI-D) **(A)**. Peak voxels that expressed decreased structural association in the PD-MCI-D group compared with the PD-MCI-ND group **(C)**. The seed or voxels are significantly correlated with neurobehavioral scores. **(B,D)** Partial regression plots with 95% confidence interval (dashed line) showing the relationships among the neurobehavioral scores, fronto-insula seed volume, and volume of peak clusters with statistical significance. GDS, Geriatric Depression Scales; VOSP, Visual Object and Space Perception Battery. Residuals are plotted for each variable to adjust for the effects of age, gender, and education.

The peak clusters showing differences in the brain volumes between the PD-MCI-ND and PD-MCI-D groups (**Figure 4A**) were positive correlated with GDS in right fusiform gyrus and left superior parietal gyrus, and positive correlated with VOSP in right hippocampus and right superior parietal gyrus (*p* < 0.05) (**Figure 4B**). Other neurobehavioral scores did not correlate with the peak clusters volume (*P* > 0.05).

For SN seed and peak clusters, we first explored whether the seed region volume was correlated with the selected cognitive test. Since age showed inverse correlation with GDS (*r* = -0.249, *P* = 0.029), verbal fluency scores in the fruit category (*r* = -0.294, *P* = 0.009) and VOSP scores (*r* = -0.367, *P* = 0.001) correlation was analyzed after controlling for age. After adequately controlling the possible covariate, the right fronto-insula seed (**Figure 4C**) volumes showed significant inverse correlation with the GDS (*r* = -0.231, *P* = 0.046) (**Figure 4D**). For the peak clusters showing differences in covariance strength between the 2 PD-MCI groups (**Figure 4C**), the left lingual gyrus volume was inversely correlated with the GDS (*r* = -0.266, *P* = 0.021), the right hippocampal volume was significantly correlated with the verbal fluency scores in the fruit category (*r* = 0.270, *P* = 0.010), and the left caudate volume was significantly correlated with VOSP scores (*r* = 0.233, *P* = 0.046) (**Figure 4D**). Other clusters volume did not correlate with neurobehavioral scores (*P* > 0.05).

## Discussion

### Main Findings

By constructing the SCN, this study provides data on the influence of SN on depressive state and severity in PD-MCI. There are three major findings. First, smaller insular seed volumes were found in the PD-MCI-D group that also correlated with the GDS. Stronger seed-peak covariance strength was observed in the PD-MCI-ND group emphasizing the importance of SN. Second, the regression model suggests that the seed or peak cluster volume depicts specific cognitive scores, especially in the insula, left lingual gyrus, left caudate, and hippocampus. Lastly, the VBM analysis reveals the differences and similarities of atrophic patterns in PD-MCI-ND and PD-MCI-D groups. However, increased atrophy in the PD-MCI-ND group and structure-neurobehavioral correlation with GDS and VOSP cognitive test suggest compensatory mechanism in PD-MCI-D. This study forges a link between SN and PD-MCI-D and also validates the data by previous PD-MCI studies (Beyer et al., 2007; Aybek et al., 2009; Jokinen et al., 2009; Martin et al., 2009; Song et al., 2011) between SN and neurobehavioral function.

### The Role of Insular Seed in PD-MCI-D

Lower insular seed was found in our PD-MCI-D patients, compared with the PD-MCI-ND. According to Braak’s staging hypothesis of PD (Braak et al., 2006), the insula is one of the vulnerable regions by alpha-synuclein deposition. Changes in the activity and volume of insula in depression suggest a critical role of this region in modulating emotional perception. Depression in patients with PD is found to correlate with reduced serotonin 1A receptor availability of the right insula in a positron emission tomography imaging study (Ballanger et al., 2012). All these evidence support the link between the right insular cortex with subjective feeling states and emotional self-awareness, which is relevant to depression (Farb et al., 2013). The novelty of the present study lies in the fact that we used the insular seed volume to depict the depression severity in patients with PD-MCI, supporting possible trajectory of alpha-synucleinopathy-related neurodegenerative processes in emotional controls. Similar to our patients with PD-MCI-D, decreased frontoinsular GM volume was found in patients with major depressive disorder (Takahashi et al., 2010).

### SN and Depressive State in PD-MCI

The structural covariance data might be considered as an indirect measurement of connectivity effectiveness. In addition to the insular seed, decreased structural association within the SN in our PD-MCI-D group provides a possible neural bridge linking key cortical hubs to depressive state in PD-MCI. Reduced BOLD between the anterior insula and ACC in patients with major depressive disorder has been reported (Veer et al., 2010). Stronger correlations may suggest greater regional connectivity and synchronized GM loss in the regions targeted by the pathological process. The findings of the PD-MCI-D group may reflect segregated and less integrated components, compared with PD-MCI-ND group in the SN that could predict depressive scores. The peak clusters showing differences in covariance strength between the two PD-MCI groups in this study included the left lingual gyrus, the right hippocampal and the left caudate.

### Role of Lingual Gyrus Region and Depression

Our result showed a clear association between the right insular seed volume and depressive severity. However, the pattern of volumetric correlation was not restricted to the insula. The cluster volume of the lingual gyrus also showed an inverse correlation with the GDS, with worse depressive scores being specifically associated with smaller cluster volume. How might larger cluster volume in the lingual gyrus contribute to better performance of depressive scores? One possibility is that individuals with larger lingual gyrus cluster volume are able to process happy faces normally. This mechanism might be supported by the functional imaging studies in patients with depression that revealed abnormal processing of happy faces in the lingual gyrus in patients with depression (Fu et al., 2007). Another possibility is that larger cluster lingual gyrus volume would have more abundant 5-HT2 receptors, supporting better neurochemical stimulation for a less depressive mood. A previous study has found that diminished 5-HT2 receptors in the lingual gyrus are correlated with depressive symptoms (Yatham et al., 2010). The lingual gyrus plays an important role in the connection between the visual pathway and limbic system (Conrad and Stumpf, 1975). Besides, a larger volume of lingual gyrus can be a predictor of early antidepressant response in patients with major depressive disorder (Jung et al., 2014). These studies suggest that functional and structural abnormality in the lingual gyrus can be a predictor of depressive severity. Taken together, our data for the first time demonstrated that both the insula and lingual seed volume were associated with depressive severity. Therefore, our finding suggests the link between the insula and lingual gyrus within the SN might be a potential network basis for depression in PD-MCI.

### Role of Other Peak Cluster – the Right Hippocampus and Left Caudate

In PD, correlations between MMSE and the density of Lewy neurites in the hippocampus and amygdala were reported (Churchyard and Lees, 1997). In this study, significant relationships between the right hippocampal volume with the generation of fruit category and the left caudate volume with VOSP were found. The finding of the relationship between fruit verbal fluency with the hippocampus has received attention before (Gleissner and Elger, 2001), and notably, is concordant with a recent study where the hippocampus was implicated in the late phase of semantic verbal fluency (Catheline et al., 2015). As our patients with PD-MCI did not perform worse than the NC on the verbal fluency test in the fruit category, the better scores are possibly dependent on the hippocampal network integrity, in contrast to the fact that early speech production is dependent on executive networks. The decreased dopamine transporter in the caudate has been reported to confer impairment of visuospatial skills (Marquie et al., 2014).

### Atrophic Patterns in PD-MCI

In VBM analysis, we found significant GM alterations in the bilateral frontotemporal and bilateral occipital regions in the PD-MCI group. The involvement of the frontotemporal cortex in PD-MCI is supported by a recent study that is related to the prediction of cognitive function (Xu et al., 2016). The significant decrease in the volume of right cerebellum in patients with PD-MCI is consistent with the literature observation that the lower GM volume in the cerebellum contributes to gait difficulty in Parkinson’s disease (Rosenberg-Katz et al., 2013). Larger GM reduction in the PD-MCI-ND group was observed in the right fusiform, right hippocampus, and bilateral superior parietal gyrus compared with the PD-MCI-D group. Studies have found that regional volume changes appear to be dynamic throughout the course of illness, with the structures being enlarged in the first period and reduced as the illness progresses. In previous studies using MRI findings, it has been showed that patients with major depressive disorder had increased GM volume in the posterior cingulate cortex, inferior frontal gyrus, and amygdala (Lorenzetti et al., 2009; Yang et al., 2015). The increased volume of the inferior frontal gyrus in major depressive disorder is positively correlated with sustained attention (Yang et al., 2015). As depression severity was relatively mild in our patients with PD-MCI, our results might suggest that the initial enlargement of the right fusiform gyrus, right hippocampus, and bilateral superior parietal gyri in the early phase of PD-MCI-D. The volume of the right hippocampus and superior parietal gyrus are positively correlated with the sustained visuospatial function of VOSP.

### Limitation

Some limitations of our study need to be addressed. First, this is a cross-sectional investigation; longitudinal follow-up is needed to observe the temporal relationship between structural association and clinical symptoms and whether the observed differences reflect transient or long-term changes, as well as the effect of larger regional volume in PD-MCI-D on longitudinal cognitive change. Second, our study relies on clinical, rather than autopsy-proven diagnosis. However, our diagnosis follows standard diagnostic criteria that have a good diagnosis accuracy based on a clinicopathological study (Hughes et al., 1992). Third, in this study, we used seed-based analysis with an emphasis on SN. Using independent component analysis or resting state function MRI data in the future may further elucidate other networks involving depression in PD-MCI. It is important to note that only very specific set of regions, selected on the basis of their demonstrating an abnormal structural association relationship, were used to evaluate correlation with our neuropsychological assessments. In other words, in the absence of concurrent structural abnormality, our clinical-pathological relation is not a retest of previous work investigating relationships among fruit verbal fluency, visuospatial function, and regional GM atrophy.

## Conclusion

Overall, this study is the first to present a picture of the structural covariance of SN in patients with PD-MCI-D and PD-MCI-ND. The results of this study suggest that the frontoinsular seed volume within the SN influences the presence and severity of depression in patients with PD-MCI. The SN might be one of the possible mechanisms underlying the neural basis of depression in patients with PD-MCI while there is strength gradient between the seed and peak clusters from non-depressive to depressive PD-MCI, thus supporting the importance of SN network alterations in PD degenerative network.

## Ethics Statement

The study was approved by Chang Gung Memorial Hospital’s Institutional Review Committee on Human Research (105-1374C, 201601682B0).

## Author Contributions

Research project conception, organization, execution: Y-TC, C-CC, C-WH, W-NC, M-KW, and S-WH. Statistical analysis execution and review and critique: J-JL and C-YL. Manuscript preparation: Y-TC, C-HL, and C-CC.

## Conflict of Interest Statement

The authors declare that the research was conducted in the absence of any commercial or financial relationships that could be construed as a potential conflict of interest.

## Footnotes

1 http://www.fil.ion.ucl.ac.uk/spm/

## References

[B1] Aarsland D. Pahlhagen S. Ballard C. G. Ehrt U. Svenningsson P. (2012). Depression in Parkinson disease–epidemiology, mechanisms and management. *Nat. Rev. Neurol.* 8 35–47. 10.1038/nrneurol.2011.189 22198405

[B2] Alexander-Bloch A. Giedd J. N. Bullmore E. (2013). Imaging structural co-variance between human brain regions. *Nat. Rev. Neurosci.* 14 322–336. 10.1038/nrn3465 23531697 PMC4043276

[B3] Amieva H. Lafont S. Rouch-Leroyer I. Rainville C. Dartigues J. F. Orgogozo J. M. (2004). Evidencing inhibitory deficits in Alzheimer’s disease through interference effects and shifting disabilities in the Stroop test. *Arch. Clin. Neuropsychol.* 19 791–803. 10.1016/j.acn.2003.09.006 15288332

[B4] Aybek S. Lazeyras F. Gronchi-Perrin A. Burkhard P. R. Villemure J. G. Vingerhoets F. J. (2009). Hippocampal atrophy predicts conversion to dementia after STN-DBS in Parkinson’s disease. *Parkinsonism Relat. Disord.* 15 521–524. 10.1016/j.parkreldis.2009.01.003 19349206

[B5] Ballanger B. Klinger H. Eche J. Lerond J. Vallet A. E. Le Bars D. (2012). Role of serotonergic 1A receptor dysfunction in depression associated with Parkinson’s disease. *Mov. Disord.* 27 84–89. 10.1002/mds.23895 21994070

[B6] Beyer M. K. Janvin C. C. Larsen J. P. Aarsland D. (2007). A magnetic resonance imaging study of patients with Parkinson’s disease with mild cognitive impairment and dementia using voxel-based morphometry. *J. Neurol. Neurosurg. Psychiatry* 78 254–259. 10.1136/jnnp.2006.093849 17028119 PMC2117633

[B7] Braak H. Bohl J. R. Muller C. M. Rub U. de Vos R. A. Del Tredici K. (2006). Stanley Fahn Lecture 2005: the staging procedure for the inclusion body pathology associated with sporadic Parkinson’s disease reconsidered. *Mov. Disord.* 21 2042–2051. 10.1002/mds.21065 17078043

[B8] Bressler S. L. Menon V. (2010). Large-scale brain networks in cognition: emerging methods and principles. *Trends Cogn. Sci.* 14 277–290. 10.1016/j.tics.2010.04.004 20493761

[B9] Brown R. Jahanshahi M. (1995). Depression in Parkinson’s disease: a psychosocial viewpoint. *Adv. Neurol.* 65 61–84. 7872153

[B10] Burn D. J. (2002). Beyond the iron mask: towards better recognition and treatment of depression associated with Parkinson’s disease. *Mov. Disord.* 17 445–454. 10.1002/mds.10114 12112190

[B11] Cardoso E. F. Maia F. M. Fregni F. Myczkowski M. L. Melo L. M. Sato J. R. (2009). Depression in Parkinson’s disease: convergence from voxel-based morphometry and functional magnetic resonance imaging in the limbic thalamus. *Neuroimage* 47 467–472. 10.1016/j.neuroimage.2009.04.059 19398020

[B12] Catheline G. Amieva H. Dilharreguy B. Bernard C. Duperron M. G. Helmer C. (2015). Semantic retrieval over time in the aging brain: structural evidence of hippocampal contribution. *Hippocampus* 25 1008–1016. 10.1002/hipo.22423 25614980

[B13] Chang C. C. Chang Y. Y. Chang W. N. Lee Y. C. Wang Y. L. Lui C. C. (2009). Cognitive deficits in multiple system atrophy correlate with frontal atrophy and disease duration. *Eur. J. Neurol.* 16 1144–1150. 10.1111/j.1468-1331.2009.02661.x 19486137

[B14] Chang C. C. Hsu J. L. Chang W. N. Huang S. H. Huang C. W. Chang Y. T. (2016). Metabolic covariant network in relation to nigrostriatal degeneration in carbon monoxide intoxication-related parkinsonism. *Front. Neurosci.* 10:187. 10.3389/fnins.2016.00187 27199649 PMC4853409

[B15] Chang C. C. Liu J. S. Chang Y. Y. Chang W. N. Chen S. S. Lee C. H. (2008). (99m)Tc-ethyl cysteinate dimer brain SPECT findings in early stage of dementia with Lewy bodies and Parkinson’s disease patients: a correlation with neuropsychological tests. *Eur. J. Neurol.* 15 61–65. 18042240 10.1111/j.1468-1331.2007.02001.x

[B16] Churchyard A. Lees A. J. (1997). The relationship between dementia and direct involvement of the hippocampus and amygdala in Parkinson’s disease. *Neurology* 49 1570–1576. 10.1212/WNL.49.6.1570 9409348

[B17] Clos M. Rottschy C. Laird A. R. Fox P. T. Eickhoff S. B. (2014). Comparison of structural covariance with functional connectivity approaches exemplified by an investigation of the left anterior insula. *Neuroimage* 99 269–280. 10.1016/j.neuroimage.2014.05.030 24844743 PMC4251452

[B18] Conrad C. D. Stumpf W. E. (1975). Direct visual input to the limbic system: crossed retinal projections to the nucleus anterodorsalis thalami in the tree shrew. *Exp. Brain Res.* 23 141–149. 10.1007/BF00235456 810358

[B19] Domellof M. E. Ekman U. Forsgren L. Elgh E. (2015). Cognitive function in the early phase of Parkinson’s disease, a five-year follow-up. *Acta Neurol. Scand.* 132 79–88. 10.1111/ane.12375 25644230

[B20] Farb N. A. Segal Z. V. Anderson A. K. (2013). Attentional modulation of primary interoceptive and exteroceptive cortices. *Cereb. Cortex* 23 114–126. 10.1093/cercor/bhr385 22267308 PMC3513954

[B21] Fu C. H. Williams S. C. Brammer M. J. Suckling J. Kim J. Cleare A. J. (2007). Neural responses to happy facial expressions in major depression following antidepressant treatment. *Am. J. Psychiatry* 164 599–607. 10.1176/ajp.2007.164.4.599 17403973

[B22] Gleissner U. Elger C. E. (2001). The hippocampal contribution to verbal fluency in patients with temporal lobe epilepsy. *Cortex* 37 55–63. 10.1016/S0010-9452(08)70557-4 11292161

[B23] Huang C. W. Tsai M. H. Chen N. C. Chen W. H. Lu Y. T. Lui C. C. (2015). Clinical significance of circulating vascular cell adhesion molecule-1 to white matter disintegrity in Alzheimer’s dementia. *Thromb. Haemost.* 114 1230–1240. 10.1160/TH14-11-0938 26289958

[B24] Hughes A. J. Ben-Shlomo Y. Daniel S. E. Lees A. J. (1992). What features improve the accuracy of clinical diagnosis in Parkinson’s disease: a clinicopathologic study. *Neurology* 42 1142–1146. 10.1212/WNL.42.6.1142 1603339

[B25] Jabbi M. Keysers C. (2008). Inferior frontal gyrus activity triggers anterior insula response to emotional facial expressions. *Emotion* 8 775–780. 10.1037/a0014194 19102588

[B26] Janvin C. C. Larsen J. P. Aarsland D. Hugdahl K. (2006). Subtypes of mild cognitive impairment in Parkinson’s disease: progression to dementia. *Mov. Disord.* 21 1343–1349. 10.1002/mds.20974 16721732

[B27] Jokinen P. Brück A. Aalto S. Forsback S. Parkkola R. Rinne J. O. (2009). Impaired cognitive performance in Parkinson’s disease is related to caudate dopaminergic hypofunction and hippocampal atrophy. *Parkinsonism Relat. Disord.* 15 88–93. 10.1016/j.parkreldis.2008.03.005 18434233

[B28] Jung J. Kang J. Won E. Nam K. Lee M. S. Tae W. S. (2014). Impact of lingual gyrus volume on antidepressant response and neurocognitive functions in Major Depressive Disorder: a voxel-based morphometry study. *J. Affect. Disord.* 169 179–187. 10.1016/j.jad.2014.08.018 25200096

[B29] Kostic V. S. Agosta F. Petrovic I. Galantucci S. Spica V. Jecmenica-Lukic M. (2010). Regional patterns of brain tissue loss associated with depression in Parkinson disease. *Neurology* 75 857–863. 10.1212/WNL.0b013e3181f11c1d 20686125

[B30] Lin P. H. Tsai S. J. Huang C. W. Mu-En L. Hsu S. W. Lee C. C. (2016). Dose-dependent genotype effects of BDNF Val66Met polymorphism on default mode network in early stage Alzheimer’s disease. *Oncotarget* 7 54200–54214. 10.18632/oncotarget.11027 27494844 PMC5342335

[B31] Litvan I. Goldman J. G. Troster A. I. Schmand B. A. Weintraub D. Petersen R. C. (2012). Diagnostic criteria for mild cognitive impairment in Parkinson’s disease: Movement Disorder Society Task Force guidelines. *Mov. Disord.* 27 349–356. 10.1002/mds.24893 22275317 PMC3641655

[B32] Lorenzetti V. Allen N. B. Fornito A. Yucel M. (2009). Structural brain abnormalities in major depressive disorder: a selective review of recent MRI studies. *J. Affect. Disord.* 117 1–17. 10.1016/j.jad.2008.11.021 19237202

[B33] Lu P. H. Edland S. D. Teng E. Tingus K. Petersen R. C. Cummings J. L. (2009). Donepezil delays progression to AD in MCI subjects with depressive symptoms. *Neurology* 72 2115–2121. 10.1212/WNL.0b013e3181aa52d3 19528519 PMC2697965

[B34] Marc L. G. Raue P. J. Bruce M. L. (2008). Screening performance of the 15-item geriatric depression scale in a diverse elderly home care population. *Am. J. Geriatr. Psychiatry* 16 914–921. 10.1097/JGP.0b013e318186bd67 18978252 PMC2676444

[B35] Marquie M. Locascio J. J. Rentz D. M. Becker J. A. Hedden T. Johnson K. A. (2014). Striatal and extrastriatal dopamine transporter levels relate to cognition in Lewy body diseases: an (11)C altropane positron emission tomography study. *Alzheimers Res. Ther.* 6:52. 10.1186/s13195-014-0052-7 25429309 PMC4245149

[B36] Martin W. R. Wieler M. Gee M. Camicioli R. (2009). Temporal lobe changes in early, untreated Parkinson’s disease. *Mov. Disord.* 24 1949–1954. 10.1002/mds.22680 19606493

[B37] Mechelli A. Crinion J. T. Noppeney U. O’Doherty J. Ashburner J. Frackowiak R. S. (2004). Neurolinguistics: structural plasticity in the bilingual brain. *Nature* 431:757. 10.1038/431757a 15483594

[B38] Menon V. (2015). “Salience network,” in *Brain Mapping: An Encyclopedic Reference* Vol. 2 ed. Toga A. W. (Cambridge, MA: Academic Press) 597–611. 10.1016/B978-0-12-397025-1.00052-X

[B39] Modrego P. J. Ferrandez J. (2004). Depression in patients with mild cognitive impairment increases the risk of developing dementia of Alzheimer type: a prospective cohort study. *Arch. Neurol.* 61 1290–1293. 10.1001/archneur.61.8.1290 15313849

[B40] Montembeault M. Rouleau I. Provost J. S. Brambati S. M. (2016). Altered gray matter structural covariance networks in early stages of Alzheimer’s disease. *Cereb. Cortex* 26 2650–2662. 10.1093/cercor/bhv105 25994962 PMC4869809

[B41] Rapport L. J. Millis S. R. Bonello P. J. (1998). Validation of the Warrington theory of visual processing and the visual object and space perception battery. *J. Clin. Exp. Neuropsychol.* 20 211–220. 10.1076/jcen.20.2.211.1169 9777475

[B42] Reginold W. Duff-Canning S. Meaney C. Armstrong M. J. Fox S. Rothberg B. (2013). Impact of mild cognitive impairment on health-related quality of life in Parkinson’s disease. *Dement. Geriatr. Cogn. Disord.* 36 67–75. 10.1159/000350032 23774742

[B43] Reitan R. M. (1955). The relation of the trail making test to organic brain damage. *J. Consult. Psychol.* 19 393–394. 10.1037/h0044509 13263471

[B44] Remy P. Doder M. Lees A. Turjanski N. Brooks D. (2005). Depression in Parkinson’s disease: loss of dopamine and noradrenaline innervation in the limbic system. *Brain* 128 1314–1322. 10.1093/brain/awh445 15716302

[B45] Rosenberg-Katz K. Herman T. Jacob Y. Giladi N. Hendler T. Hausdorff J. M. (2013). Gray matter atrophy distinguishes between Parkinson disease motor subtypes. *Neurology* 80 1476–1484. 10.1212/WNL.0b013e31828cfaa4 23516323 PMC3662357

[B46] Seeley W. W. Crawford R. K. Zhou J. Miller B. L. Greicius M. D. (2009). Neurodegenerative diseases target large-scale human brain networks. *Neuron* 62 42–52. 10.1016/j.neuron.2009.03.024 19376066 PMC2691647

[B47] Seeley W. W. Menon V. Schatzberg A. F. Keller J. Glover G. H. Kenna H. (2007). Dissociable intrinsic connectivity networks for salience processing and executive control. *J. Neurosci.* 27 2349–2356. 10.1523/JNEUROSCI.5587-06.2007 17329432 PMC2680293

[B48] Song S. K. Lee J. E. Park H. J. Sohn Y. H. Lee J. D. Lee P. H. (2011). The pattern of cortical atrophy in patients with Parkinson’s disease according to cognitive status. *Mov. Disord.* 26 289–296. 10.1002/mds.23477 21370255

[B49] Spreen O. Strauss E. (1998). “Geriatric depression scale (GDS),” in *A Compendium of Neuropsychological Tests* eds Spreen O. Strauss E. (New York, NY: Oxford University Press) 612–616.

[B50] Takahashi T. Yucel M. Lorenzetti V. Tanino R. Whittle S. Suzuki M. (2010). Volumetric MRI study of the insular cortex in individuals with current and past major depression. *J. Affect. Disord.* 121 231–238. 10.1016/j.jad.2009.06.003 19540599

[B51] Veer I. M. Beckmann C. F. van Tol M. J. Ferrarini L. Milles J. Veltman D. J. (2010). Whole brain resting-state analysis reveals decreased functional connectivity in major depression. *Front. Syst. Neurosci.* 4:41 10.3389/fnsys.2010.00041 PMC2950744 20941370

[B52] Xu Y. Yang J. Hu X. Shang H. (2016). Voxel-based meta-analysis of gray matter volume reductions associated with cognitive impairment in Parkinson’s disease. *J. Neurol.* 263 1178–1187. 10.1007/s00415-016-8122-3 27113603

[B53] Yang X. Ma X. Huang B. Sun G. Zhao L. Lin D. (2015). Gray matter volume abnormalities were associated with sustained attention in unmedicated major depression. *Compr. Psychiatry* 63 71–79. 10.1016/j.comppsych.2015.09.003 26555494

[B54] Yatham L. N. Liddle P. F. Lam R. W. Zis A. P. Stoessl A. J. Sossi V. (2010). Effect of electroconvulsive therapy on brain 5-HT(2) receptors in major depression. *Br. J. Psychiatry* 196 474–479. 10.1192/bjp.bp.109.069567 20513859

[B55] Zaki J. Ochsner K. N. Hanelin J. Wager T. D. Mackey S. C. (2007). Different circuits for different pain: patterns of functional connectivity reveal distinct networks for processing pain in self and others. *Soc. Neurosci.* 2 276–291. 10.1080/17470910701401973 18633819 PMC2913618

[B56] Zimmerman M. Emmert-Aronson B. O. Brown T. A. (2011). Concordance between a simpler definition of major depressive disorder and diagnostic and statistical manual of mental disorders, fourth edition: an independent replication in an outpatient sample. *Compr. Psychiatry* 52 261–264. 10.1016/j.comppsych.2010.07.009 21497219

